# How do contextual factors influence naloxone distribution from syringe service programs in the USA: a cross-sectional study

**DOI:** 10.1186/s12954-023-00755-4

**Published:** 2023-02-28

**Authors:** Barrot H. Lambdin, Lynn Wenger, Ricky Bluthenthal, Tyler S. Bartholomew, Hansel E. Tookes, Paul LaKosky, Savannah O’Neill, Alex H. Kral

**Affiliations:** 1 RTI International, Berkeley, CA USA; 2 University of California San Francisco, San Francisco, CA USA; 3 University of Washington, Seattle, WA USA; 4 University of Southern California, Los Angeles, CA USA; 5 University of Miami, Miami, FL USA; 6 North American Syringe Exchange Network, Tacoma, WA USA; 7 National Harm Reduction Coalition, Oakland, CA USA

**Keywords:** Syringe services programs, Opioid overdose, Naloxone distribution, Implementation science

## Abstract

**Background:**

Naloxone is a medication that can quickly reverse an opioid overdose. Syringe service programs (SSPs) are community-based prevention programs that provide a range of evidence-based interventions in the USA, including naloxone distribution. Attributes of SSPs make them ideal settings for naloxone distribution—they have staff and delivery models that are designed to reach people who use drugs where they are. We assessed which outer and inner setting factors of SSPs were associated with naloxone distribution in the USA.

**Methods:**

We surveyed SSPs in the USA known to the North American Syringe Exchange Network in 2019. Using the exploration, preparation, implementation and maintenance framework, we assessed inner and outer contextual factors associated with naloxone distribution among SSPs (*n* = 263 or 77% of SSPs). We utilized negative binomial regression to assess which factors were associated with the number of naloxone doses distributed and people receiving naloxone.

**Results:**

SSPs reported distributing 710,232 naloxone doses to 230,506 people in the prior year. Regarding outer setting, SSPs located in areas with high levels of community support had a higher level of naloxone distribution (aIRR = 3.07; 95% confidence interval (CI): 2.09–4.51; *p* < 0.001) and 110% (*p* = 0.022) higher rate of people receiving naloxone (aIRR = 2.10; 95% CI 1.46–3.02; *p* < 0.001) in the past 12 months. The legal status of SSPs and the level of need was not significantly associated with naloxone distribution. Regarding inner setting, SSPs with proactive refill systems (aIRR = 2.08; 95% CI 1.27–3.41; *p* = 0.004), greater number of distribution days (aIRR = 1.09 per day; 95% CI 1.06–1.11; *p* < 0.001) and older programs (aIRR = 1.06 per year; 95% CI 1.02–1.11; *p* = 0.004) were associated with higher levels of naloxone distribution. Also, SSPs with proactive refill systems (aIRR = 2.23; 95% CI 1.38–3.58; *p* = 0.001); greater number of distribution days (aIRR = 1.04; 95% CI 1.02–1.07; *p* < 0.001) and older programs (aIRR = 1.11; 95% CI 1.05–1.17; *p* < 0.001) were associated with a higher number of people receiving naloxone.

**Conclusion:**

We identified outer and inner setting factors of SSPs that were associated with greater naloxone distribution. It is critical to ensure SSPs are adequately resourced to build community support for services and develop service delivery models that maximize naloxone distribution to address the nation’s opioid overdose crisis.

## Background

Opioid-involved overdose mortality has increased more than six-fold since 1999 with particularly steep increases in recent years [1–5]. Between 2013 and 2019, the age adjusted rate of deaths involving synthetic opioids other than methadone increased by 1040% [4]. April of 2021 marked the first time in the USA when over 100,000 people died from an overdose in the prior 12 months [6].

Opioid-related overdose deaths can be prevented with naloxone—a medication that can quickly reverse the respiratory depression that can be induced by opioids during an overdose [7]. Since the probability of permanent injury or death increases with the amount of time a person remains in respiratory depression, the timelines of naloxone administration is critical [8]. Increasing access to naloxone in high-risk communities and training laypeople—people who use drugs (PWUD), their family members and peers—about overdose identification and naloxone administration increases the possibility that naloxone is effectively deployed to prevent opioid overdose fatalities [9, 10].

Between 1996 and 2014, a national study found that over 80% of laypeople who participated in community-based naloxone distribution were PWUD and over 80% of reported overdose reversals were performed by PWUD [16]. A recent analysis from Washington estimated that 99% of overdoses in the state were reversed by laypeople, and 1% were reversed by law enforcement personnel [11]. These findings emphasize the importance of expanding and scaling-up access to overdose education and naloxone distribution (OEND) among PWUD.

Syringe service programs (SSPs) are community-based prevention programs that provide access to sterile syringes and injection equipment [12]. In the late 1980s, SSPs were first implemented to respond to the HIV epidemic in the USA. Over the last 30 years, a robust body of research has shown that SSPs are safe, effective and cost-effective at preventing HIV among PWID [13]. In addition, SSPs are ideal organizations for implementing other types of evidence-based interventions for PWUD, including HIV and HCV testing, and TB screening [14, 15]. Attributes of SSPs that make them favorable environments for offering services to PWUD include their location in community-based settings which are easily accessible for their participants; their staff’s culturally competency in providing services with dignity and respect; and their ongoing relationships with participants who trust them to care for their health. As such, SSPs are well positioned to provide other evidence-based interventions, including overdose education and naloxone distribution (OEND). Specific to naloxone distribution, SSPs reach people who are at high risk of observing or experiencing opioid overdose and who are involved in the large majority of overdose reversals [11, 12].

While many SSPs pioneered naloxone distribution in the USA, naloxone distribution has not reached sufficient levels of saturation at the community level [6, 16, 17]. A recent study of people who inject drugs (PWID) found that 49% had witnessed and 16% had experienced at least one overdose in the past six months—yet only 35% currently possessed naloxone [18]. For naloxone to effectively prevent opioid overdose fatalities, a higher proportion of potential bystanders need to be knowledgeable about and consistently possess, carry and use naloxone [9]; and ensuring naloxone is present during drug use events is critical [19]. Yet, persistent systems and organizational-level barriers, including negative public attitudes around the utility of naloxone, discrimination associated with substance use and unsupportive legal and funding environments—limit naloxone distribution [20, 21].

The exploration, preparation, implementation and sustainment (EPIS) framework highlights key phases of the implementation process and identifies factors across and within the outer and inner context that can influence the implementation process of evidence-based interventions [22–24]. The outer context represents factors external to the organization that can either support or slow implementation, such as federal, state, county or local policies; funding; or community environment. The inner context refers to characteristics within the organization, such as leadership, staffing, organizational culture and climate [24]. No studies have examined how aspects of the outer and inner context of SSPs are related to increased levels of naloxone distribution. To fill this gap in the literature, we carried out a cross-sectional study to examine which outer and inner context factors of SSPs lead to greater naloxone distribution.

## Methods

### Study setting

Operating since 1989, the North American Syringe Exchange Network (NASEN) has served as a clearinghouse for syringes, supplies, technical assistance and other materials for SSPs throughout the USA. Over the past 30 years, NASEN has served as one of the premier networks and resource centers throughout the USA for SSPs to share knowledge, lessons learned and promotion of best practices. NASEN has one of the most comprehensive datasets of SSPs operational in the USA with (1) the Directory database—SSPs that would like to be listed on their public facing directory; and (2) the Buyers Club database—SSPs that purchase supplies through NASEN at discounted pricing.

### Study population

Our study population included syringe service programs operating throughout the USA in 2019. SSPs were defined as community-based prevention programs that provide access to drug use supplies, including sterile syringes and injection equipment. Though not a requirement, SSPs often provide a range of other services to improve the health of people who use drugs, including overdose education and naloxone distribution; drug testing; vaccinations; testing and linkage to medical care; and other social support services. All SSPs operating within the USA, Territories and Tribal Nations were eligible to participate in our survey. We asked the executive director, or their designee, of the organization to participate in the study by providing information about their SSP.

### Study procedures

We conducted an online, cross-sectional survey of SSPs throughout the USA from February to June of 2019. The overall goal of the survey was to characterize the outer and inner context of SSPs and their relationship with naloxone distribution. The North American Syringe Exchange Network (NASEN) maintains a database and online directory of SSPs throughout the country which contains contact information and basic organizational characteristics. NASEN contacted all SSPs from their database with an initial email to request their participation in an online survey. Two follow-up emails were sent 2 and 4 weeks after the initial email to encourage participation. For organizations that did not respond to email requests, additional follow-up was conducted with phone calls and one-on-one emails to ensure they had received the communication and could troubleshoot any barriers to their participation. For participating SSPs, the first page of the online survey summarized our study procedures; noted that participation was voluntary; described our provisions to maintain privacy; detailed our reimbursement for their participation; and asked for their consent to participate before they could move forward to provide responses to survey questions. In addition, we used data from the National Center for Health Statistics with regard to the population size and number of opioid overdose deaths in 2017.

Our study procedures were reviewed by internal review board within the Office of Research Protection at RTI International’s internal review board and was determined to be exempt under 45 CFR 46.104 (d)(2)(ii). Yet, the study did include a consent process which disclosed the activities of the research, the procedures to be performed, that participation was voluntary, the investigator name and contact information and the provisions to maintain the privacy of subjects. Because the study was determined to be exempt, the consent process did not require a written or electronic signature or a waiver of written documentation of consent. However, participants had to indicate their consent to participate by selecting a button on the webpage before advancing into the online survey.

### Measures

Measures that were included in this study were informed by the exploration, preparation, implementation and maintenance (EPIS) framework [24]. The EPIS framework highlights the importance of the relationship of the outer and inner contextual factors of organizations with implementation of evidence-based interventions. For our study, we wanted to assess which characteristics from SSPs’ outer and inner context were associated with levels of naloxone distribution. Our study outcomes included the number of naloxone doses distributed by the SSP in the prior 12 months as well as the number of people receiving naloxone from the SSP in the prior 12 months. Prior research has shown that increases in the number of people receiving naloxone and the number of naloxone doses distributed were significantly associated with reductions in opioid overdose mortality in the surrounding community [25–27].

Our exposure variables included measures from the outer and inner context of SSPs. With regard to the outer context, we asked each SSP about the legal status of their organization—whether they operated in a sanctioned or unsanctioned environment; and how they characterized levels of community support for their program on a scale of 0–100. Community support was dichotomized at the median of the distribution—a local community support rating of 80 out of 100—to allow for enough data to make a comparison and provide a meaningful cut-off point. To estimate the level of need in the surrounding community, we used the opioid overdose mortality rate in the counties where the SSP operated. Collected from the National Center for Health Statistics [5], these data were geocoded to the county level and matched to the county where each of the SSPs operated. If an SSP operated across multiple counties, the number of opioid overdose deaths and the population size were aggregated for all the counties in which they operated. Opioid overdose mortality rates were identified using ICD-10 codes X40–X44; X60–X64; X85; or Y10–Y14, where the multiple cause of death codes included T40.0, T40.1, T40.2, T40.3, T40.4 or T40.6. We created a 3-level ordered categorical variable of low (≤ 10), medium (> 10–< 18) and high (≥ 18), split at the tertile of the distribution.

With regard to the inner context, we asked each SSP how sustained their OEND implementations were by asking about number of years since they implemented OEND; whether the SSP regularly asked participants if they needed a naloxone refill (proactive) compared to waiting for participants to ask them for a refill (passive); and the number of days of service the SSP provides OEND to their participants in a typical 28-day (4 week) period.

### Statistical analysis

We used negative binomial regression models to estimate the association between inner and outer contextual factors on the number of people receiving naloxone and the number of naloxone doses, separately. Models accounted for population size in the county or counties where the SSP operated. We modeled outer and inner contextual factors separately. For each set of analyses, we built full, adjusted models to generate adjusted incident rate ratios (aIRR), 95% confidence intervals (CI) and associated *p* values. We also carried out a sensitivity analysis; first, we log transformed our outcome variables to approximate a normal distribution; then, we utilized linear regression with robust variances to build similar models. All analyses were conducted using Stata (v16.1).

## Results

A total of 263 SSPs responded to our survey, representing 77% of the 342 known SSPs operating in 2019. Among the responding SSPs, 247 (94%) indicated that they distributed naloxone, and 237 (96%) of those distributing naloxone provided information with regard to the number of naloxone doses distributed and the number of people receiving naloxone in the prior year. On a scale of 1–100 (with 100 representing high support), the reported median level of community support was 80; the median opioid overdose mortality rate in the surrounding community was 14 per 100,000 people; and most SSPs provided OEND services at least 15 of the last 28 days. In total, SSPs reported distributing 710,232 naloxone doses to 230,506 people in the prior year; per SSP, median annual number of naloxone doses distributed was 880 and median annual number of people receiving naloxone was 350 (Table 1).

**Table 1:** Outer and inner context of participating syringe service programs (SSP) in the USA (*N* = 263)

SSP Legal Status, *n* (%)	
Sanctioned	220 (92)
Unsanctioned	19 (8)
Community support for naloxone^a^, median (IQR)	80 (70–90)
Opioid overdose death rate (per 100,000), median (IQR)	14 (8–26)
Refill system, *n* (%)	
Passive	38 (16)
Proactive	199 (84)
Days of service, median (IQR)	15 (6–20)
Age of program, median (IQR)	2 (1–5)
Naloxone doses distributed, median (IQR)	880 (200–2236)
People receiving naloxone, median (IQR)	350 (77–1000)

^a^Respondents were asked to characterize the local community support for OEND on a scale of 1–100

Regarding the outer context, the legal status of SSPs was not associated with rates of naloxone doses distributed or people receiving naloxone. In addition, the opioid overdose death rate in the counties where the SSP operated was not associated with rates of naloxone doses distributed or people receiving naloxone. However, SSPs located in areas with high levels of community support had a 207% (*p* < 0.001) higher rate of naloxone distribution and 110% (*p* = 0.022) higher rate of people receiving naloxone (Table 2).

**Table 2:** Multivariable model of outer context factors and naloxone distribution from syringe services programs (SSP)

	Naloxone doses distributed		People receiving naloxone	
	aIRR (95% CI)	*p* value	aIRR (95% CI)	*p* value
SSP legal status				
Sanctioned	(ref)		(ref)	
Unsanctioned	1.24 (0.70, 2.21)	0.454	1.38 (0.81, 2.36)	0.238
Community support for naloxone				
< 80	(ref)		(ref)	
≥ 80	3.07 (2.09, 4.51)	< 0.001	2.10 (1.46, 3.02)	< 0.001
Opioid overdose death rate				
Low (≤ 10)	(ref)		(ref)	
Medium (> 10–< 18)	1.23 (0.77, 1.98)	0.381	1.00 (0.65, 1.55)	0.990
High (≥ 18)	1.22 (0.77, 1.94)	0.392	1.18 (0.77, 1.83)	0.441

Both models account for population size in the counties where SSP operates

Regarding the inner context, SSPs with proactive refill systems had a 207% (*p* < 0.001) higher rate of naloxone distribution and 110% (*p* = 0.022) higher rate of people receiving naloxone. SSPs with a greater number of naloxone distribution days had a 207% (*p* < 0.001) higher rate of naloxone distribution and 110% (*p* = 0.022) higher rate of people receiving naloxone. SSPs that had been implementing OEND for a longer period of time had a 207% (*p* < 0.001) higher rate of naloxone distribution and 110% (*p* = 0.022) higher rate of people receiving naloxone (Table 3). Findings from our sensitivity analyses aligned with these results.

**Table 3:** Multivariable model of inner context factors and naloxone distribution from syringe services programs (SSP)

	Naloxone doses distributed		People receiving naloxone	
	aIRR (95% CI)	*p* value	aIRR (95% CI)	*p* value
Refill system				
Passive	(ref)		(ref)	
Proactive	2.08 (1.27, 3.41)	0.004	2.23 (1.38, 3.58)	0.001
Days of service	1.09 (1.06, 1.11)	< 0.001	1.04 (1.02, 1.07)	< 0.001
Sustained implementations	1.06 (1.02, 1.11)	0.004	1.11 (1.05, 1.17)	< 0.001

## Discussion

This study fills an important gap in the scientific literature with regard to understanding relationships of outer and inner contextual factors of SSPs with naloxone distribution. Our most striking finding was that opioid overdose mortality rates in the prior year were not a significant predictor of naloxone distribution. This is particularly concerning as the opioid overdose mortality rates from the previous year are perhaps our best available measure for how much naloxone distribution is needed in a given area. Yet, these findings are consistent with prior research showing that the number of PWID with HIV was not predictive of SSP services [28, 29]. Ideally, higher levels of naloxone distribution would occur where the need is higher, and this finding suggests that the systems that support community-based naloxone—legal environment, funding climate; local, state and federal support; etc.—are not generating adequate responses to the overdose crisis at the local level [30].

With respect to outer context, our results showed that SSPs with higher levels of community support in surrounding areas reported higher levels of naloxone distribution. Our findings mirror qualitative findings that demonstrate the pivotal role that surrounding communities can play either through support or resistance [31]. Taken together, these findings point to the importance of community engagement focused on building a broader understanding of the benefits of community-based naloxone and addressing concerns within the community [32]. Yet, our data suggest that SSPs in many parts of the country face high levels of community opposition; for them to carry out their public health services effectively, more resources are needed to strengthen support within the community. However, which approaches are effective at improving community support remains an open area of inquiry for future research.

Our results also showed that unsanctioned SSPs had similar levels of naloxone distribution compared to those operating in sanctioned environments. Prior research has shown that policy/legal environments are important predictors of whether community-based naloxone programs or buprenorphine treatment within SSPs are implemented [33, 34]. Therefore, while the legal environment can facilitate the establishment of programs, our finding suggests that a sanctioned environment is insufficient to yield greater levels of naloxone distribution from SSPs. While it is encouraging that a group of highly motivated people can achieve naloxone distribution on par with SSPs that are operating in a sanctioned environment, this dynamic is also concerning. In a well-functioning system of community-based naloxone, it is reasonable to expect that a sanctioned SSP could gain access to more financial and human resources, thereby translating to higher levels of naloxone distribution. Our findings suggest that this is not the case.

Supporting programs to increase their days of service and supporting them into sustainment is important for building larger-scale naloxone distribution. While our study did not collect information about funding levels, recent studies have shown that the median annual budget of SSPs was $85,000 in 2020 [33, 35]. This estimate is far below recommended funding levels for SSPs; a recent analysis estimated essential costs ranging from $400,000 for a rural SSP serving 250 clients to $1.9 million for an urban SSP serving 2500 clients [36]. Insufficient funding levels for SSPs could be influencing our finding that most SSPs only operated 15 days of OEND services in the past 28 days. Increasing funding levels of these programs as well as strengthening administrative systems to absorb resources from a variety of different sources is critical.

Our finding that proactive refill systems were associated with higher levels of naloxone distribution highlight an important, modifiable organizational characteristic. A recent initiative highlighted that strengthening refill systems can be a critical component to improving the number of doses and people with naloxone in the community [37]. SSPs have ongoing interactions with their participants; this makes them a great programmatic setting for initial OEND engagement and to provide ongoing support and refills of naloxone. In addition to building community, financial and legal support, providing technical assistance to SSPs to co-create efficient OEND screening and refill systems for their participants can help SSPs optimize their impact with participants.

For this study, the EPIS framework was an important tool to strengthen our understanding of organizations’ outer and inner contextual factors and their relation to the scale of naloxone distribution [22]. A recent qualitative study also used the EPIS framework to explore challenges SSPs faced during the COVID pandemic as they made programmatic adaptations to prevent overdose deaths while simultaneously keeping workers and participants safe from COVID. This study identified themes within the outer context (service environment/policies, funding and interorganizational environment and networks), inner context (organizational and individual characteristics) and innovation factors (evidence-based practice characteristics) as important to sustaining implementation of naloxone distribution at the onset of COVID [38]. In a separate study, the EPIS framework was used to understand implementation determinants for medications for opioid use disorder within prison and jails, also identifying outer context (legal environment, funding and interorganizational environment and networks) and inner context (individual characteristics, staffing, training) as important drivers to implementation [39]. Overall, EPIS can be a useful framework to understand implementation determinants as well as the implementation process for evidence-based interventions that can reduce deaths due to opioid involved overdoses.

It is important to consider potential methodological limitations with our study. We are unable to generalize to all SSPs in the USA as we do not know the full universe of SSPs. Since 1992, NASEN has served as the primary resource for SSPs in the country. NASEN operates a buyers club for SSPs to acquire supplies for harm reduction services, offers free supplies to new and resource-constrained SSPs, and maintains a database of all known SSPs in the USA. We utilized this information to get in touch with SSPs for our survey, but it remains possible that other SSPs are unknown to NASEN. Since 23% of SSPs did not complete the survey, there is also the possibility of response bias. It is particularly plausible that underfunded or smaller SSPs had a lower response rate [40]. In addition, SSP data could contain mistakes because information was self-reported and not validated with official records by the study team. Finally, there are potential misclassification errors with the publicly available data of overdose deaths.

## Conclusion

This study identified outer and inner setting factors were associated with greater naloxone distribution from syringe service programs. With rising opioid overdose mortality rates for over 2 decades, the need to address these factors is of paramount importance, and past due. Ensuring that SSPs operate within supportive legal and community environments; receive sufficient and sustained financial support and build strong delivery systems is vital to maximize naloxone distribution in such a way that effectively addresses the nation’s opioid overdose crisis.

## Data Availability

The datasets used and/or analyzed during the current study are available from the corresponding author on reasonable request.

## Abbreviations

SSP: Syringe service programs
OEND: Overdose education and naloxone distribution
PWUD: People who use drugs
PWID: People who inject drugs
EPIS: Exploration, preparation, implementation and sustainment
aIRR: Adjusted incidence rate ratio
CI: Confidence interval
